# Simple and Effective Secure Group Communications in Dynamic Wireless Sensor Networks

**DOI:** 10.3390/s19081909

**Published:** 2019-04-22

**Authors:** Hisham N. AlMajed, Ahmad S. AlMogren

**Affiliations:** Chair of Cyber Security, Department of Computer Science, College of Computer and Information Sciences, King Saud University, Riyadh 11633, Saudi Arabia; 438105079@student.ksu.edu.sa

**Keywords:** group key, group key management, secure group communication, wireless sensor networks

## Abstract

Wireless Sensor Network (WSN) is a growing area of research in terms of applications, life enhancement and security. Research interests vary from enhancing network performance and decreasing overhead computation to solving security flaws. Secure Group Communication (SGC) is gaining traction in the world of network security. Proposed solutions in this area focus on generating, sharing and distributing a group key among all group members in a timely manner to secure their communication and reduce the computation overhead. This method of security is called SGC-Shared Key. In this paper, we introduce a simple and effective way to secure the network through Hashed IDs (SGC-HIDs). In our proposed method, we distribute a shared key among the group of nodes in the network. Each node would have the ability to compute the group key each time it needs to. We provide a security analysis for our method as well as a performance evaluation. Moreover, to the best of our knowledge, we present for the first time a definition of joining or leaving attack. Furthermore, we describe several types of such an attack as well as the potential security impacts that occur when a network is being attacked.

## 1. Introduction

Wireless Sensor Network (WSN) brings new ideas and innovations to our life [1,2,3]. Today, we are surrounded by a wide range of applications and opportunities. For instance, healthcare wearable devices, smart environment sensors, agriculture sensors and military devices are examples of such important applications [4,5,6,7,8]. These devices and sensors aim to collect data to provide a numerous of valuable results. However, these low end devices come with very limited computation and processing capabilities [9,10,11]. Therefore, to overcome this issue, there is a need for a remote unit with computation capability to perform such a process.

Furthermore, these devices are small and have limited internal power source (e.g., batteries). Thus, they need to be power efficient to reduce power consumption while monitoring and gathering data to maximize their battery life [12,13,14,15]. In fact, power consumption and data transmission reduction are affected by several areas in the network, for instance network topology, device architecture, data gathering scheme and optimized security schemes [16,17,18,19].

In addition, these networks consist of many nodes communicating with each other [20] to sense, collect, process, and transmit event specific information to accomplish certain task [21]. Networks can be static (closed to certain nodes) or dynamic (open), which allows nodes to freely join and leave the network. Static networks are less vulnerable to security breaches than dynamic networks. For instance, Mutual Authentication is a key part in dynamic networks than static networks. In addition, the computations needed in dynamic networks allow nodes to freely join and leave the network are bigger than the computations needed in static networks.

WSNs have several types of communications based on their topology. For instance, nodes within the group communicate with each other, nodes communicate with the BS and the BS communicates with all nodes (broadcast). Thus, securing the communication in WSNs is affected by communication type and the cryptography solution used can vary. For example, a shared group key is used to secure broadcast of messages between BS and all nodes in the group, which reduces the overhead of creating multiple ciphered texts for each node, thus decreasing the node computation and power consumption. Moreover, it is necessary that the cryptography methodology implemented fits the ability of the nodes to reduce node computation overhead. For instance, Elliptic Curve Cryptography (ECC) is a better choice to used to secure communications for low capability nodes than RSA [22,23,24].

To receive collected data from low-end devices, these devices need to communicate in an insecure environment [25,26,27,28] (such as the Internet) or insecure infrastructure (public cloud) [29] to send data to a high-end unit for further computation and processing. A common network model to connect devices consists of three main ways [30,31,32,33]. The first is the communication between each node and remote unit, which is used for mutual authentication and to exchange collected data. The second is the communication between two nodes in the same group, where some applications force nodes to exchange data. The third is the broadcast message from remote unit (such as BS) to all nodes within its group for routine announcements or to broadcast nodes joining or leaving the group. Therefore, security becomes an important challenging in designing those networks [34,35,36,37].

In this paper, we discuss WSNs types, security flaws with each type and the cryptography methodology used to secure these networks. The remainder of this paper is organized as follows. In Section 2, we briefly discuss the related works. In Section 3, we define several network models. In Section 4, we list the important security requirements for these models. In Section 5, we show the proposed SGC-HIDs method in general and in WSNs specifically. Finally, in Section 6, we conclude and show future work.

## 2. Related Works

Tan et al. [38] proposed secure and efficient certificateless authentication scheme for VANETs. Their scheme consists of three phases to provide secrecy to the group communications. The first phase is registration phase, where a vehicle registers with a trusted party in offline mode. The second phase is the authentication phase, where the vehicle is authenticated based on the offline registration phase. The third is the shared key between the group produced using Chinese Remainder Theorem (CRT). The same authors [39] proposed generating a shared key by deriving it from pre-defined parameters between RSU and the vehicle instead of using CRT. He et al. [40,41] proposed CLPA scheme to secure group communications with eight polynomial-time algorithms. Their scheme is based on the certificateless approach to mutually authenticate a user and a key generation center. The shared key used for decrypting a broadcast message is not part of this scheme.

Seo et al. [42] provided a new scheme to secure communication in dynamic WSNs. In their proposed scheme, the authors secured communication between BS and nodes using four types of keys. The first key, Certificateless Public/Private Key, is used to generate a mutually authenticated and pairwise key between a new node and the BS. The second key, is used to secure each node’s private communication with the BS each with an individual node key. The third key, Pairwise Key, is used to secure communication between each node in the cluster. Finally, the cluster key (the group key) is used to broadcast messages to all nodes within s cluster. The drawback of this scheme in terms of SGC is that it needs to generate a secret key for the cluster each time a node joins or leaves, which creates extra overhead. In addition, the cluster head will broadcast a new shared secret key and after hash it, using each node key in the network. This leads to increased network utilization each time a node joins or leaves the group, which could generate an extensive attack lead to make WSN unavailable.

Gupta and Biswas [43] proposed a group key by asking each node in the group to produce certain values. These values can be computed by each node to generate the group key for the group. The main advantage is that this procedure generates a highly trusted group key for the group without the need of trusted third party. However, this scheme is inefficient when the number of nodes is increased. In addition, as each node is asked to produce its own values, it leads to increasing the node computational requirements. Moreover, this scheme does not discuss the node ability to join or leave the WSN and its effects on regenerating the group key for the remaining nodes within the group.

Tan and Chung [44] proposed an effective scheme to SGC and generate a shared group key. In their scheme, a trusted key generator is responsible for generating a group key where each node in the group can derive the shared secret key from it using CRT. In addition, the authors discussed the joining or leaving procedures and stated that, with each joining or leaving, there is a need to regenerate the group key. Their scheme is secure and efficient, however it needs to be applied on powerful devices (PCs) capable of doing extensive computation that low power devices cannot. Moreover, their scheme does not cover the backward secrecy, as a leaving node still has the shared secret key.

Guo et al. [45] proposed a self-healing group key distribution protocol. In their protocol, each node receives a random t-degree polynomial, a multiplicative group of finite field of two orders and a unique identity for each node. For each new node joining the group also receives the same parameters excluding the previous session keys to keep backward secrecy. This protocol reduces the key distribution overhead efficiently, however it has several drawbacks. For instance, the authors stated that a joining node in separate sessions increases the computation and network overhead, thus it is better to group all new nodes in one joining session to overcome the overhead computation and reduce network utilization.

AlShammari and Elliethy [46] proposed a key distribution protocol for WSNs. Their protocol aims to distribute a symmetric key to a group of nodes to secure its communications. Symmetric cryptography is faster and uses less computation than asymmetric cryptography, therefore they preloaded shared public key to all nodes before network implementation. Thus, each node uses this group key to secure the distribution of group secret key. These steps provide a light and feasible scheme that distributes group key, however these steps introduce some vulnerabilities, such as group key leaking when node a stolen and it is vulnerable to forward and backward secrecy. In addition, the authors used RSA in their protocol, thus it could be enhanced by using ECC as it uses smaller encryption key with same encryption strength as RSA with large key.

Perrig et al. proposed a classic broadcast authentication protocol μTESLA [47], which is based on TESLA protocol but improved to fit for unreliable network. The main idea of TESLA protocols is to divide the distribution keys for each encrypted communications. After an encrypted packet broadcasts to all nodes, the corresponding key to decrypt the packet is sent during an interval of time. Thus, it is crucial to keep nodes in synchronization time with the cluster head to avoid losing the corresponding key during the interval time. Huang et al. [48], improved μTESLA by replacing the interval time to divide and release the corresponding key using Queuing Theory. They claimed that the fixed time interval in original μTESLA could increase unnecessarily a node’s computation. Therefore, they replaced the fixed time interval by data flow interval. The authors claimed they effectively improved the efficiency of the utilization of keys and reduced the network communication overhead and computational cost. However, they did not discuss the forward and backward secrecy for joining or leaving nodes.

## 3. Wireless Sensor Networks Models

Nodes on a network can be distributed and communicate in many ways. Node distribution over the network defines its topology into several types, such as ring, star, mesh and cluster [49]. Moreover, these networks can also be categorized as either static (closed) or dynamic (open) [21], which defines the WSN’s orientation. In static network, nodes cannot freely join or leave the existing group. Therefore, when implemented, all nodes are fixed and connected to the network [50]. This type of network provided less security vulnerability, as all nodes are defined prior to the network implementation.

The second type of network allows nodes to freely join or leave the group, where this main aspect defines it as a dynamic network [51]. Dynamic WSNs need to address security flaws that can lead to compromising the security of the network. For instance, it allows new nodes to join the network, which raises the need to check the legitimacy of that node to be part of the group. In addition, a leaving node could still leak useful information that leads to an adversary breaching the security of the network (such as leaking the group key). Thus, forward and backward secrecy need to be considered when designing dynamic WSNs.

## 4. WSNs Security Requirements

In this section, we discuss security requirements for WSNs. These requirements are affected by design constraints, network performance requirements and security aspects. For instance, according to the authors of [52,53], AES-128 needs 1.66 ms to encrypt 29 bytes of data and 2.12 ms to decrypt the same data. Similarly, the time needed to hash the same data using SHA-1 function is roughly 1.62 ms. Therefore, these computation requirements need to be fit for low power nodes and the encryption scheme should be applicable for less computational devices (e.g., ECC). Moreover, forward and backward secrecy should be considered when setting up dynamic network. Table 1 illustrates four requirements that need to be considered to implement successful WSNs.

### 4.1. Static WSNs Security Requirements

WSNs need to connect to insecure network to accomplish their tasks [54]. This connection increases the security vulnerabilities that need to be considered when implementing such a network. For instance, a Man-In-The-Middle attack (MITM) is one of the many security issue in networks where an adversary is set in the middle of two parties’ communication and listens to confidential data [55]. In addition, the topology may increase network security vulnerabilities, for instance the dynamic network may be vulnerable to impersonation attack where an adversary node acts as s legitimate node and becomes part of the group [56].

Furthermore, WSNs are vulnerable to other security flaws. In insecure physical environments, a node can be stolen and modified to be an adversary node [57]. Therefore, the integrity of the network needs to be maintained and act instantly to any unauthorized modification to it. Equally important to outside attacks, inside attacks need to be addressed and considered while designing new networks. An example of an inside attacks is unauthorized access to data that have been gathered and collected by the nodes. To address this issue, an access control scheme needs to be set up to prevent such security breaching [58]. However, securing WSNs and implementing access control will not guarantee a network security. An authorized user could intentionally leak data and share them with unauthorized parties. To address this issue, a Data Leakage Prevention (DLP) scheme handles this kind of security flaws.

**Figure 1:**
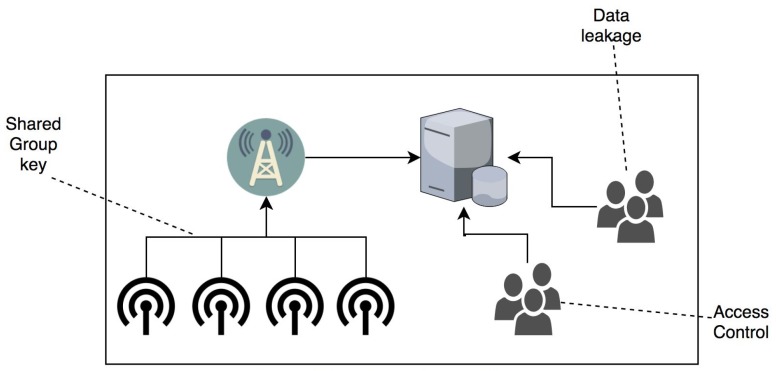
High level security vulnerabilities in static WSNs.

Figure 1 depicts high level security vulnerabilities in static WSNs.

### 4.2. Dynamic WSNs Security Requirements

As described in the previous section, dynamic WSNs are vulnerable to the same security flaws as static WSNs. In addition, they are also vulnerable to more security flaws as nodes can freely leave and join the group [59]. Nodes leaving the network can reveal current information about shared secrets that leads to forward secrecy attack. Equally important, nodes joining the network can also lead to another attack if they can gain any data that have been sent before joining the network, which is known as backward secrecy attack.

Furthermore, in dynamic WSNs, node authentication can prevent any security attacks that lead to compromising the network. Nodes without proper authentication could lead to several attacks, for instance impersonation attack and man in the middle attack [60]. Thus, it is necessary to mitigate this vulnerability by implementing strong Mutual Authentication (MA) to properly authenticate nodes and the BS [61]. Furthermore, all BSs with the network and the main server should also be authenticated to overcome any unauthorized access to collected data.

**Figure 2:**
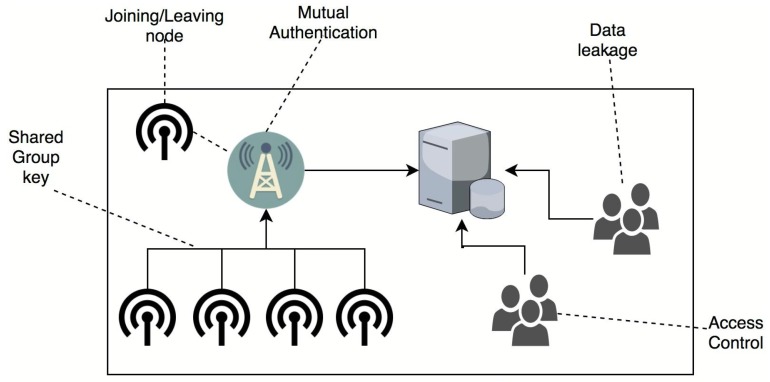
High level security vulnerabilities in dynamic WSNs.

Figure 2 depicts high level security vulnerabilities in dynamic WSNs.

### 4.3. Secure Group Communication in WSNs

SGC schemes are classified into three categorizes: centralized, contributory, and hybrid [59]. Centralized means that nodes depend on a trusted party (such as base station) to generate and distribute all required keys. Contributory means that all nodes collaborate for the management of the group rather than depend on a third party. Hybrid means that the generation and distribution of all required keys are the responsibility of a third party as well as all nodes in the group.

The main issue with the first category (centralized) is that it will make WSN fall into bottleneck, as this trusted party is a standalone device, which could be compromised or become unavailable. This consideration is valid and acceptable, however, in such network, we are counting on the base station to gather data from the nodes within the group. Thus, we can also add the initiating of the shared key for the group to be the base station’s responsibility.

In this paper, we focus on dynamic networks using hybrid schemes only, as the static network’s security requirements are part of these networks. Furthermore, our main contribution is providing a novel way to secure group communications in WSNs. In addition, we discuss some schemes used to secure group communications, describe the potential security issues related to them, and compare then with SGC-HIDs.

## 5. Network Model and Proposed Scheme

**Figure 3:**
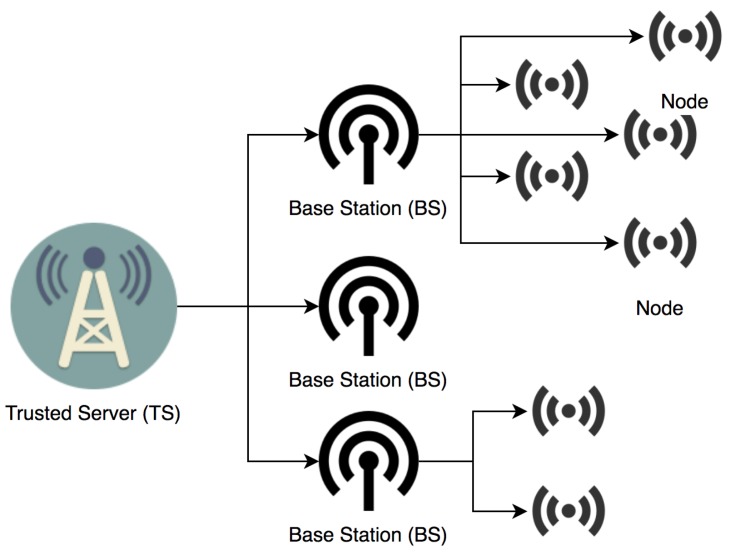
High level of network model in SGC-HIDs.

In Figure 3, we describe the high level of our network model. Each BS will handle a group of nodes that transmit data to the BS. Then, the BS transmits these data to Trusted Server (TA). As it is a dynamic WSN, any node is free to move to another group of nodes. We assume that communication between the BS and node to exchange keys is done in a secure way. Similarly, securing communication between two nodes in the group is out of our scope in SGC-HIDs. BS frequently communicates with group nodes through hello message or beacon message to prevent any unusual behavior with group nodes.

We propose SGC-HIDs to secure group communication by generating shared key using node IDs. As described above, WSNs have three types of communication:

- BS communicate with a node to exchange keys, where we assume in our scheme that it is done in a secure way.
- Nodes communicate with other nodes, where some nodes may need to communicate with a neighbor node directly. In our scheme, we do not cover this type of communication.
- The BS broadcasts messages to all group nodes, which we describe below as SGC-HIDs in our new scheme.

SGC-HIDs aims to secure the third way in WSNs only, and it consists of the following four steps:

- BS generates node parameters and the initial group key.
- Node joins the group.
- Node leaves the group.
- Node is identified as a compromised node.

### 5.1. BS Generating the Initial Group Key

When a WSN is initialized, the BS needs to generate certain values to secure network communications. These values are:

- uList contains all nodes hashed IDs ⊕ PRK.
- rList contains all nodes that leaved WSN or identified as compromised node.
- Private Random Key (PRK) is the key used to ⊕ with BS and nodes IDs before hashing. This is a necessary step as hashing ID directly would result in each node being able to determine its hashed ID. Thus, we need to prevent a node from knowing a key element in generating shared key in SGC-HIDs.
- The initial random group key consists of the hash value of ⊕ two values, where the first value is the BS ID and the second value is PRK.

### 5.2. Node Joining the Group

**Figure 4:**
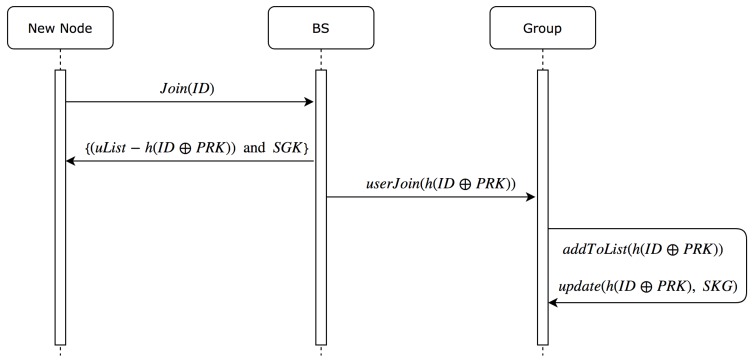
Sequence diagram for joining node steps.

In this section, we describe the procedure to share group’s key to a new node. In addition, this procedure includes the update of group key to all current nodes in the group. As stated above, we assume that a new node has already exchanged the keys with BS in a secure way. In Figure 4, we describe the sequence of steps to share and update group key.

A new node sends its ID to BS to join the group. BS sends back the uList of current nodes (except the new node itself) and new group key (current group key ⊕ hashed (new node id ⊕PRK)). Simultaneously, BS broadcasts the new joining node hashed (new node id ⊕PRK) to all current nodes in the group using old group key (before ⊕ with new hashed ID ⊕PRK). Afterwards, each node will decrypt the received broadcast using the current group key and then add the new received value to its current uList and update current group key by ⊕ it with the new received value.

Algorithms 1 describes the steps needed for a new node to join the group. The input of this algorithm is node ID and the output is the new group key and updated uList.


**Algorithm 1: joiningNode**
  **Input**: $NodeIdentifier(ID_{i})$  **Output**: {$uList™h(ID_{i}),group\;key$} _**1**_
$Check\;the\;ID_{i}\;in\;the\;rList$; _**2**_
$Node\;ID_{i}\;\leftarrow \;\left\{uList,\;groupkey⊕h\left(IDi⊕PRK\right)\right\}$; _**3**_
$uList\leftarrow uList+h(ID_{i}⊕PRK)$; _**4**_
$Group\;nodes\leftarrow h(ID_{i}⊕PRK)$;

Algorithm 2 describes how to add a new received value to the uList. Each node in the group will receive a broadcast from BS containing a new node hashed ID ⊕ PRK encrypted by the old group key to prevent the new node from receiving this value. Then, each node will decrypt the message and add it to its uList with its sequence reference.


**Algorithm 2: addToList**
  **Input**: $HashedNodeIDentifierh(ID_{i}⊕PRK)$  **Output**: $uList+h(ID_{i}⊕PRK)$ _**1**_
$Check\;the\;h(ID_{i}⊕PRK)\;in\;the\;uList$; _**2**_
Notinthelistproceedtostep3elseproceedtostep4; _**3**_
$uList\leftarrow uList+h(ID_{i}⊕PRK)$; _**4**_
BS←error;

Algorithm 3 describes how each node will update the group key. Each node will update the group key by ⊕ current group key with new received hashed ID ⊕PRK.


**Algorithm 3: updateSGK**
  **Input**: $Groupkey,h(ID_{i}⊕PRK)$  **Output**: Newgroupkey _**1**_
$group\;key\leftarrow group\;key⊕h(ID_{i}⊕PRK)$;

### 5.3. Node Leaving the Group

**Figure 5:**
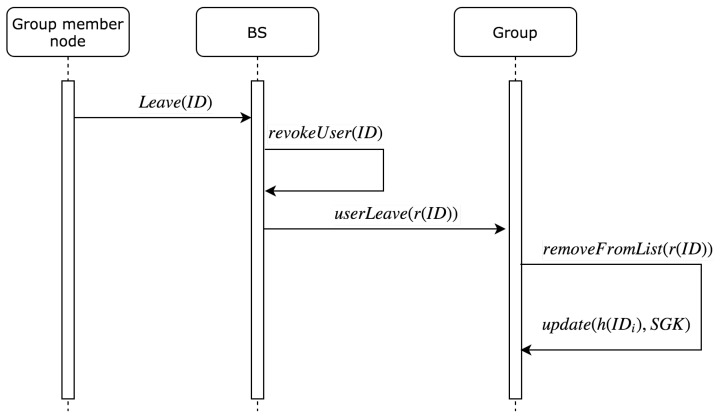
Sequence diagram for leaving node steps.

In this section, we describe the steps needed to update the group key when a node leaves the group. In addition, this section includes the steps needed to update the uList in BS and in all nodes within the group to remove the leaving node’s reference ID. Figure 5 describes the sequence of steps to update the group key and remove the reference ID from uList when the current node leaves the group.

When a node leaves the group, it will be identified as LeavedNode. This node will be added to rList with interval timeout to prevent joining or leaving attack. Defining the timeout depends on the WSN configuration, where it could be fixed time or changeable with some nodes.

Algorithm 4 describes the steps needed to update the group key when a node leave the group. BS will update the rList by adding the leaving node’s hashed ID with interval timeout to prevent joining or leaving attack. Similarly, BS will remove the reference ID of leaving node from uList to maintain the group key with all nodes.


**Algorithm 4: revokeNode**
  **Input**: $Reference\;Node\;IDentifier(ID_{i})$  **Output**: $uList™r\left(ID_{i}\right),rList+ID_{i},new\;group\;key$, _**1**_
Ifthisnodeisnotidentifiedascompromisednode→gottostep3; _**2**_
$rList\leftarrow rList+r(ID_{i})+tag\;comp$; _**3**_
$rList\leftarrow rList+r(ID_{i})$; _**4**_
$uList\leftarrow uList™r(ID_{i})$; _**5**_
$Group\;nodes\leftarrow r(ID_{i})$; _**6**_
$Group\;key\leftarrow group\;key⊕h(ID_{i}⊕PRK)$;

Algorithm 5 describes the steps for each node in the group to update the group key when a node leave the group. BS will broadcast the reference ID of the leaving node to all nodes in the group including the leaving node itself. This message is encrypted with the current group key used in the group. Each node including the leaving node will be able to decrypt the message and update the group key by ⊕ the current group key with the value matching the received reference ID from uList. However, the leaving node will not be able to update the group key as its hashed ID ⊕PRK is not included in its uList.


**Algorithm 5: removeFromList**
  **Input**: $ReferenceNodeIDentifier(ID_{i})$  **Output**: $uList™r(ID_{i})$ _**1**_
$uList\leftarrow uList™r(ID_{i})$;

### 5.4. Node Identified as Compromised Node

In some cases, a node may be identified as a compromised node. This type of nodes needs to be added to rList tagged as a compromised node to prevent it from rejoining the network. In addition, a network issue or physical attack could lead to the BS not being able to communicate with some nodes through hello message or beacon message. Therefore, BS will consider this node as compromised node for security reasons. Once the node is identified as a compromised node, BS will revokeNode and broadcast a request to all nodes to removeFromList and updateSGK with reference ID included in this request.

**Figure 6:**
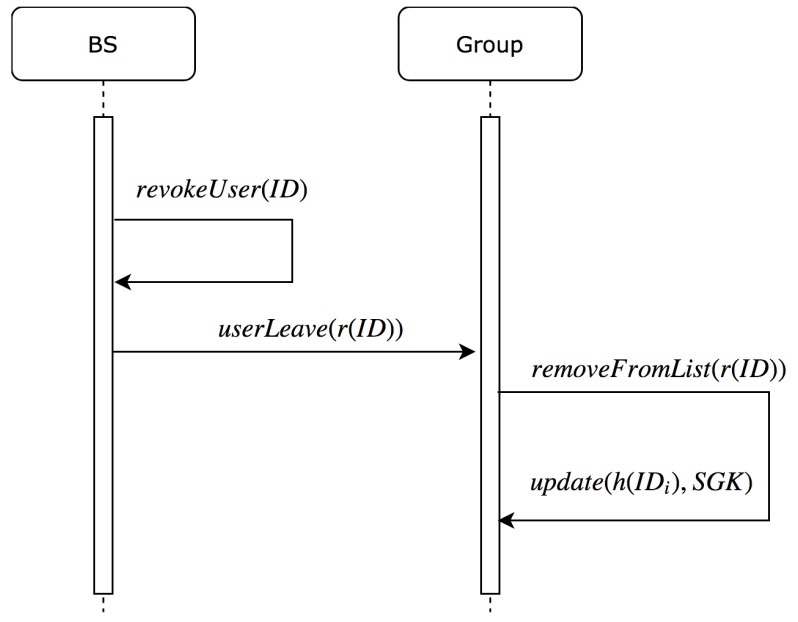
Sequence diagram for node identified as compromised node.

Figure 6 describes the sequence of steps to update the group key when a node identified as compromised node. It is clearly noted that this action is initiated by the BS to maintain the group secrecy. Therefore, all algorithm will be same as listed above.

## 6. Security Analysis

In this section, we analyze the security properties of SGC-HIDs. Security properties include: user anonymity, forward secrecy, backward secrecy, impersonation attack, and key freshness.

### 6.1. Forward Secrecy

Forward secrecy is the property that a compromise of the long-term keys used for authentication does not compromise the session keys for past connections [62]. The shared key used by nodes in the group is derived from BS key and all other nodes hashed IDs. Benefiting from one-time pad (OTP), forward secrecy in SGC-HIDs is valid to this security property. When a node requests to join the group, BS will add its hashed ID to its uList and update the group key by applying the following equation:

(1) $$groupkey⊕h(ID_{i}⊕PRK)$$

Simultaneously, BS will broadcast nodeJoin with hashed ID of joining node to the group. Therefore, all nodes except the joining node will receive the hashed ID. Thus, joining node will not be able to revert to old shared key as it does not have its hashed ID.

### 6.2. Backward Secrecy

Backward secrecy is the property that the disclosure of the responder’s private key (or any session key) does not compromise the secret key negotiated from later runs [63]. The shared key used by nodes in the group is derived from BS key and all other nodes hashed IDs. Benefiting from one-time pad (OTP), SGC-HIDs is valid to this security property. When a node requests to leave the group or is identified as a compromised node, BS will update the uList and update the group key by applying the following equation:

(2) $$group\;key⊕h(ID_{i}⊕PRK)$$

Simultaneously, BS will broadcast nodeLeave with reference ID of leaving node to all nodes in the group. All nodes except the leaving node will do the same equation as the BS using the reference ID. However, the leaving node will not be able to locate its reference ID in its uList as it is not included when this node joins the group for the first time. Thus, it will not be able to decrypt any future data broadcast by BS.

### 6.3. Key Freshness

It is highly recommended that the secret key is changed frequently. In SGC-HIDs, this security property is valid. For instance, the group key changes when a new node joins the group. In addition, for any leaving node or any node identified as a compromised node, BS and nodes update the group key.

### 6.4. Impersonation Attack

Impersonation attack means an adversary may pretend to be a legitimate user. The adversary may use a legitimate user ID and password to ask the BS to join the network. In SGC-HIDs, we assume that the keys exchanged between BS and nodes are provided in a secure way (first type of communication). However, if an adversary node is not authenticated, then it will not be able to receive the group key or generate it without the current uList and current group key.

### 6.5. User Anonymity

User anonymity aims to protect user’s privacy without breaching the system security. In SGC-HIDs, we assume that BS is a trusted party and it is responsible for all communications and data collections from all nodes in the group. Therefore, a node ID will only be exposed to the BS while all other nodes will get only its hashed ID. In addition, each node will be given the corresponding reference to leaving node to update the group key.

### 6.6. Physical Attack

In some case, a physical attack (DoS, jamming, cloning or tampering with the node) cloud lead to node not being able to communicate with the BS. As stated above, a BS will identify any node that has any issue with communication as a compromised node. When a node does not respond to a beacon message from the BS, this lead to the possibility that the node has been physically attacked. Thus, it will be added to rList with comp tagging to prevent it from join the group again.

### 6.7. Joining or Leaving Attack

To our knowledge, no one has discussed joining or leaving attack as a special type of exhausting attack. In this type of attack, an adversary could intercept the communication between BS and a legitimate node, which forces the BS to consider this node as a leaving node. However, the adversary then allows this node to communicate again with the network where the BS reinitializes a new SGC for the group and increases the BS overhead if the process happens with small interval of time. Moreover, repeating this attack could make this legitimate node be considered a compromised node and the BS would permanently disallow it from joining the network.

In addition, the legitimate node could be compromised and send request to leave the group. According to most schemes, the BS will initiate nodeLeave process. Later, the same node will ask the BS to join the network again. Similarly, the BS will initiate nodeJoin process. In most schemes, nodes compute the group key, therefore repeated joining or leaving process would make the nodes busy with this computation.

Therefore, in SGC-HIDs, any legitimate node leaving the network or unable to communicate with the BS will not be able to join the network for a fixed interval. In addition, any compromised node will be permanently removed from the network and will not be able to connect to the network.

## 7. Performance Analysis

In this section, we show a comparison between SGC-HIDs and other schemes. Although these schemes provide secure communication in WSNs between BS and node, node to node and SGC using shared key, our comparison only focused on the performance of SGC using shared key only. In addition, the comparison between these scheme is difficult for two reasons. First, these schemes provide results for the overall end-to-end encryption process, therefore their results include several phases that are not related to our interested area. Secondly, the authors of these schemes did not provide the datasets used in their evaluation to help us in the comparison step. Therefore, we extracted the steps for SGC using shared key phase from each scheme and we abstracted it to several algorithms to calculate computation time complexity. In addition, from each algorithm, we extracted four computation types, namely Generating Parameters (GP), Encryption (E), Hashing (H) and Decryption (D), which highly affect the computation of each scheme.

### 7.1. Comparison Based on SGC Features

In this section, we present the main SGC features that each scheme supports. Table 2 shows each scheme and the supported features needed in SGC.

### 7.2. Comparison Based on Computation Time Complexity

In this section, we present computation time complexity for each scheme. Table 3 show computation time complexity for SGC-HIDs in comparison with other schemes. Time complexity computation indicates how many nodes need to be updated for each processing in each phase to complete the phase. For instance, if a node leaves a group and the scheme sends an encryption message to each node using its private key, then the BS will process *n* times encryption computation O(n). Similarly, if the scheme proposes to encrypt the message by the shared key, then the BS will process one time computation O(1).

Seo et al.’s algorithm for SGC while initializing the group is:

 _**1**_
BS:Generategroupkeybyhashing;
 _**2**_
BS:EncryptgroupkeyforeachNnodes;

 _**3**_
Node:Eachnodewilldecryptgroupkey;

 _**4**_
Node:Eachnodewillvalidategroupkeybyhashing;

 _**5**_
Node:EachnodewillencryptACK;

 _**6**_
Node:EachnodewillhashACK;

 _**7**_
BS:WilldecryptACK;

 _**8**_
BS:WillvalidateACKbyhashing;

Seo et al.’s scheme does the same steps for each node joining or leaving the group.

Tan and Chung’s algorithm for SGC while initializing the group is:

 _**1**_
BS:Computenodesderivationkeys;

 _**2**_
BS:ForeachnodesBSsendsderivationkeys;

 _**3**_
BS:GenerateCRTbasedgroupkey;

 _**4**_
BS:Encryptgroupkey;

 _**5**_
BS:Signencryptedgroupkey;

 _**6**_
Node:Eachnodewillverifysignedgroupkey;

 _**7**_
Node:Eachnodewilldecryptgroupkey;

 _**8**_
Node:Eachnodewillcomputegroupkey;

Tan and Chung’s scheme only broadcasts any node joining or leaving the group to all nodes in the group.

Guo et al.’s algorithm for SGC while initializing the group is:

 _**1**_
BS:Generatesthesecretpolynomialandotherparametersaccording;

 _**2**_
BS:Constructmultiplehashingkeysmessage;

 _**3**_
BS:Encryptthehashedkeyusinghashedrandomparameter;

 _**4**_
BS:Encryptthekeysessionusinghashedrandomparameter;

 _**5**_
Node:Eachnodewilldecrypttwoencryptedkeys;

 _**6**_
Node:Eachnodewillcomputetwohashedfunctions;

Guo et al.’s scheme does the same steps for each node leaving the group. However, for the new node joining the group, it only broadcasts one request for the new node to all nodes in the group.

### 7.3. Comparison Based on Network Utilization

**Figure 7:**
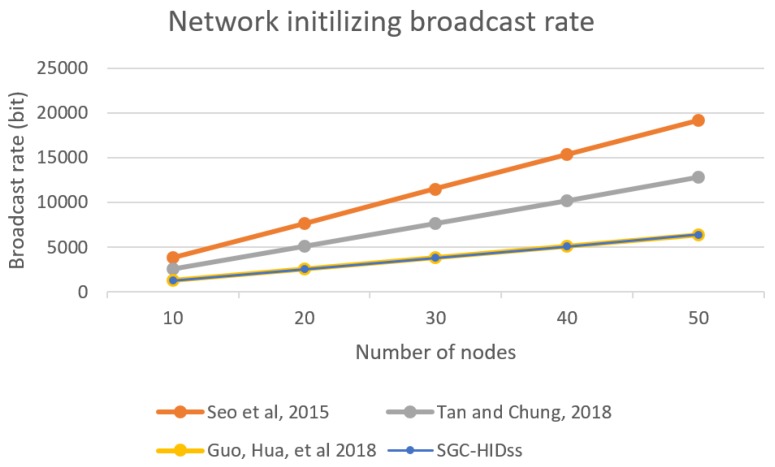
BS broadcast rate for initializing the group.

In this section, we present network utilization rates for each scheme. In this comparison, the encryption used was AES encryption with 128-bit block size. Figure 7 show utilization for SGC-HIDs in comparison to other schemes when initializing the network. It describes the packets rate growth in comparison with the growth of number of nodes in the network.

**Figure 8:**
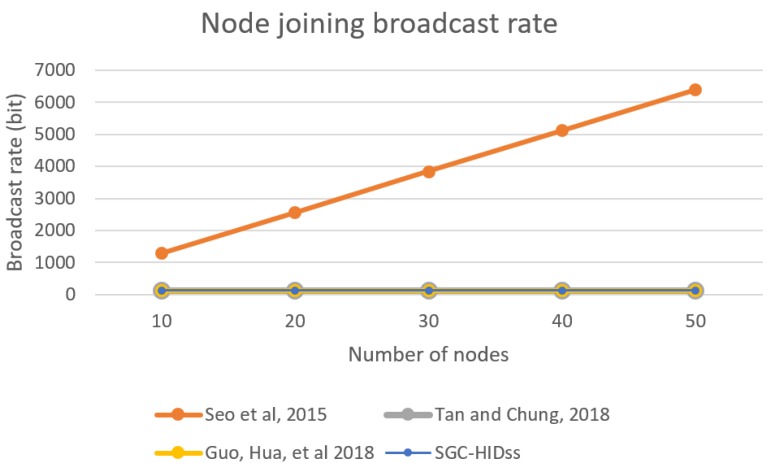
BS broadcast rate for each node joining the group.

Figure 8 shows network utilization for SGC-HIDs in comparison to other schemes when a new node joins the network. It describes the packets rate growth for each joining request in comparison with the growth of number of nodes in the network.

**Figure 9:**
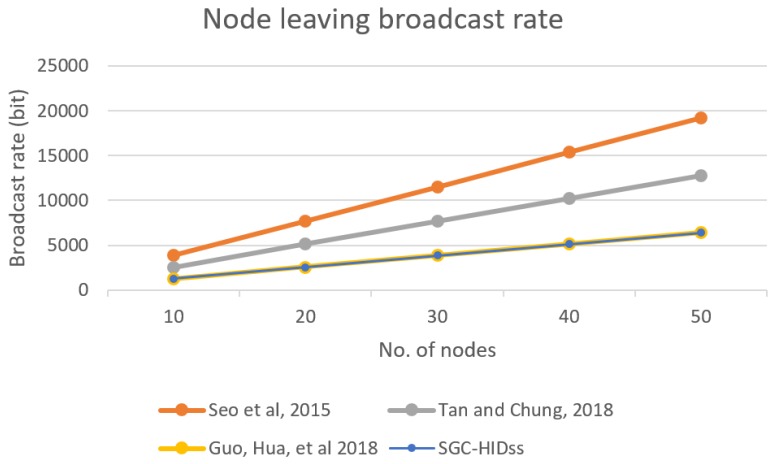
BS broadcast rate for each node leaving the group.

Figure 9 shows network utilization for SGC-HIDs in comparison to other schemes when initializing the network. It describes the packets rate growth for each leaving request in comparison with the growth of number of nodes in the network.

The time needed to encrypt, decrypt and hash for each scheme is presented below. As mentioned above, AES-128 needs 1.66 ms to encrypt 29 bytes of data and 2.12 ms to decrypt the same data. Similarly, the time needed to hash the same data using SHA-1 function is roughly 1.62 ms. However, we considered the time needed to generate system parameters; since there are no studies for such requirements, we assumed that the time needed for generating parameters was 1 ms.

**Figure 10:**
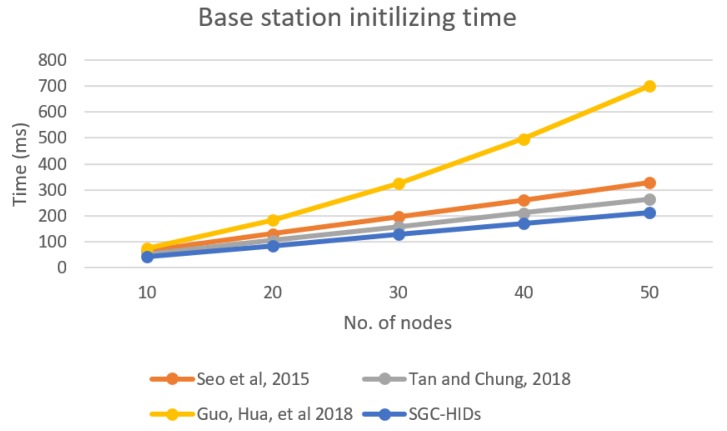
Base station computation time needed for initializing the group.

By analyzing each scheme, we found that for Seo et al. the BS will do two hashing and two encryption operations (2H+2E). In addition, each node will also do two hashing and two decryption operations (2H+2E). Similarly, Guo et al’s BS does one generating and *j* times hashing (*j* is session counter) to generates key sequence by using hash function plus two encryption operations (1H∗j+2E). In addition, node will do two hashing plus two decryption for each request (2E+2D). Finally, Tan and Chung have two generates for parameters, one encryption and one hashing operation (2GP+1E+1H). In addition, for each node, there will be one hashing and one decryption operation (1D+1H). Based on previous equations for each scheme, Figure 10 shows performance comparison for BS computation for each scheme when initializing the network. It describes the time needed to initialize the network for each scheme in comparison with the growth of number of nodes in the network.

**Figure 11:**
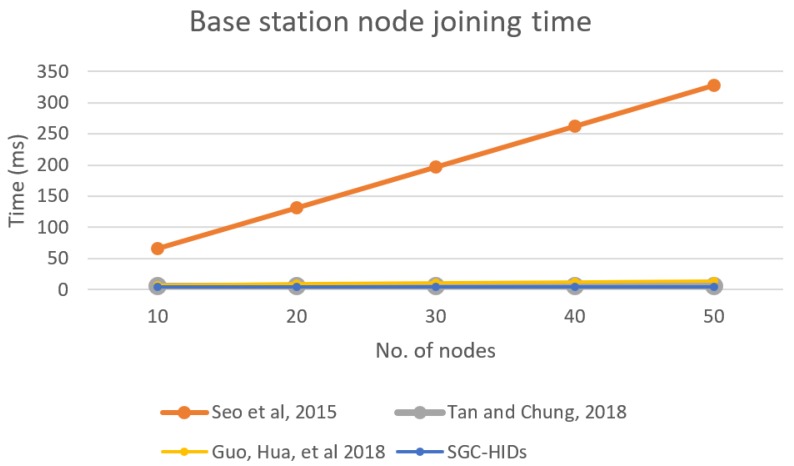
Base station computation time needed for node joining the group.

Similarly, we compute the performance for BS when new nodes join the group, and Figure 11 shows performance comparison BS for each scheme when a new node joins the network. It describes the BS time needed to initiate the joining request and update the group key for all nodes in the network for each scheme in comparison with the growth of the number of nodes in the network.

**Figure 12:**
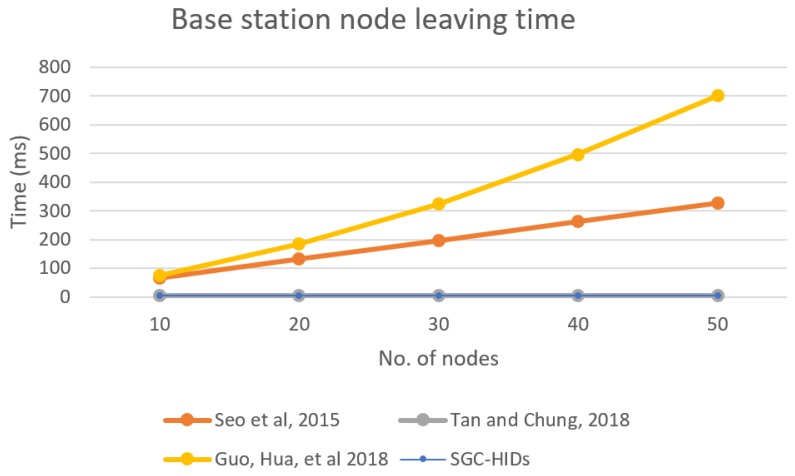
Base station computation time needed for node leaving the group.

As before, we computed the performance for BS when a group node leaves the group, and Figure 12 shows the performance comparison for BS for each scheme when a node leaves the network. It describes the BS time needed to initiate the leaving request and update the group key for all nodes in the network for each scheme in comparison with the growth of number of nodes in the network.

**Figure 13:**
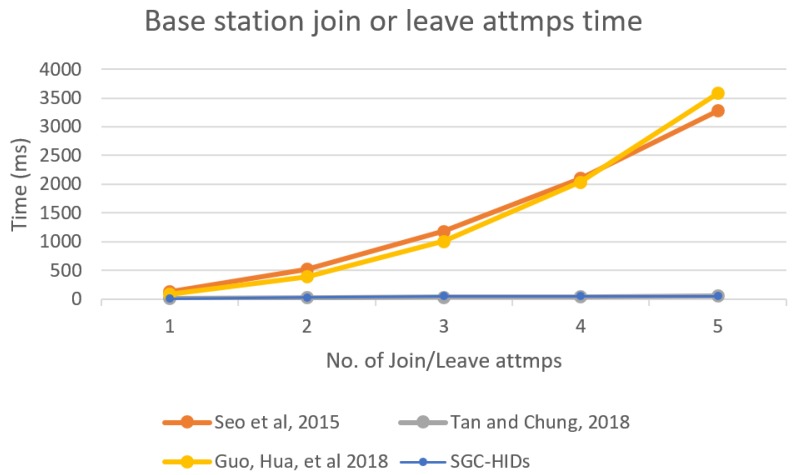
Base station computation time needed for each node joining or leaving the group.

Finally, we computed the performance for BS when joining or leaving attack id started, and Figure 13 shows the performance comparison of BS for each scheme when there is a joining or leaving attack on the network. It describes the BS computation time that occurred when such attack started in the network for each scheme to the number of attempts for join and leave to the network.

**Figure 14:**
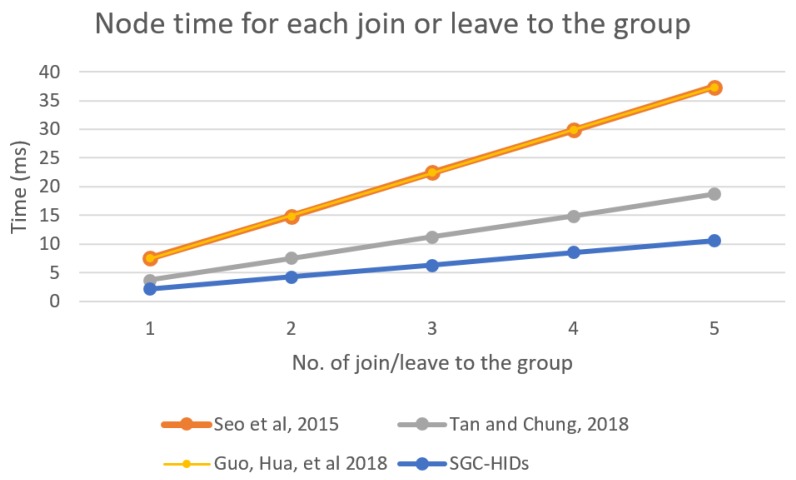
Each group nodes computation time needed for each join or leave to the group.

We computed the performance of node group when number of nodes join or leave the group. Figure 14 shows the performance for each node in the group for each scheme when nodes join or leave the group. It describes the node computation time that occurred for all requests for each scheme to the number of attempts for join and leave to the network.

## 8. Conclusions and Further Work

In this paper, we introduce SGC-HIDs, a new scheme to secure group communication (SGC) based on hashed IDs of all nodes in the group to generate shared key. In addition, we set several algorithms to describe steps needed to secure the SGC. Moreover, we introduce a description for joining or leaving attack that, to the best of our knowledge, is the first definition of such an attack. In addition, we discuss the security analysis and security attacks for the SGC-HIDs. We show that SGC-HIDs outperforms other schemes in terms of BS computation time needed for the network initialization, joining and leaving processes. Furthermore, SGC-HIDs has less node computation time for each request received for node joining or leaving. Finally, SGC-HIDs resists joining or leaving attack.

As future work, we plan to formally apply this scheme using ECC and define all scheme parameters and detail all needed steps. In addition, we will include all types of communications that used in WSNs.

## Figures and Tables

**Table 1:** General constraints for encryption in WSNs.

No	Constraint	Solution	Advantages	Disadvantages
1	Key escrow	Certificateless	More secure	Need more of computation
		Key Generator	Faster and generate secure key	Depend on third party
2	WSN	Open (Dynamic)	Scalable and efficient	Vulnerable to several security issues
		Closed (Static)	Less vulnerability to security issues	Limited scalability
3	Nodes	Low end	Cheap and used in many applications	Low computation resources
		High end	High computation resources	Expensive and limited usage
4	Restriction	High	Needed in some applications	Need more resources
		Low	Open schemes and resources	Used in fewer applications

**Table 2:** Main features in SGC.

	1	2	3	4	5	6	7	8	9
Seo et al. [42]	Y	Y	Y	N	Y	Y	Y	N	Y
Gupta and Biswas [43]	N	Y	Y	N	Y	Y	Y	N	N
Tan and Chung [44]	Y	Y	Y	N	Y	Y	N	Y	Y
Guo et al. [45]	Y	Y	Y	N	Y	Y	Y	Y	N
AlShammari and Elliethy [46]	N	N	N	Y	N	N	N	N	N
Huang et al. [48]	Y	N	Y	Y	Y	N	N	Y	Y
SGC-HIDs	Y	Y	Y	Y	Y	Y	Y	Y	Y

1, Dynamic WSNs; 2, Node compute SGC; 3, Freshness key; 4, Low Network and Computation overhead; 5, Resistance physical attack; 6, Froward Secrecy; 7, Backward Secrecy; 8, Resistance to joining or leaving Attack; 9, Scalability.

**Table 3:** Computation time complexity for each phase in each scheme.

	Initializing	Node Joining	Node Leaving
Seo et al. [42]	O(n)	O(n)	O(n)
Tan and Chung [44]	O(n)	O(1)	O(1)
Guo et al. [45]	O(n)	O(1)	O(n)
SGC-HIDs	O(n)	O(1)	O(1)

## Abbreviations

The following abbreviations are used in this manuscript:

SGC	Secure Group Communications
WSNs	Wireless Sensor Networks
BS	Base Station
CRT	Chinese Remainder Theorem
ECC	Elliptic Curve Cryptography
SGC-HIDs	Secure Group Communications using Hashed IDs
